# Primary Hyperparathyroidism in Pregnancy: Literature Review of the Diagnosis and Management

**DOI:** 10.3390/jcm10132956

**Published:** 2021-06-30

**Authors:** Dalal S. Ali, Karel Dandurand, Aliya A. Khan

**Affiliations:** Division of Endocrinology and Metabolism, McMaster University, Hamilton, ON L8S 4L8, Canada; d_alali@hotmail.com (D.S.A.); karel.dandurand@usherbrooke.ca (K.D.)

**Keywords:** hyperparathyroidism, pregnancy, calcium homeostasis, parathyroidectomy, gestational hyperparathyroidism, cinacalcet, nephrolithiasis, preeclampsia, hypercalcemia

## Abstract

Background: Parathyroid disease is uncommon in pregnancy. During pregnancy, multiple changes occur in the calcium regulating hormones which may make the diagnosis of primary hyperparathyroidism more challenging. Close monitoring of serum calcium during pregnancy is necessary in order to optimize maternal and fetal outcomes. In this review, we will describe the diagnosis and management of primary hyperparathyroidism during pregnancy. Methods: We searched MEDLINE, CINAHL, EMBASE and Google scholar bases from 1 January 1990 to 31 December 2020. Case reports, case series, book chapters and clinical guidelines were included in this review. Conclusions: Medical management options for primary hyperparathyroidism during pregnancy are severely limited due to inadequate safety data with the various potential therapies available, and surgery is advised during the 2nd trimester of pregnancy in the presence of severe hypercalcemia (calcium adjusted for albumin greater than 3.0 mmol/L (12.0 mg/dL)). Hypercalcemia should be avoided during pregnancy in order to minimize maternal and fetal complications.

## 1. Introduction

Primary hyperparathyroidism (PHPT) is a relatively common endocrine disorder and represents the most common cause of hypercalcemia in the non-pregnant population [1]. The prevalence of PHPT in pregnancy was examined in one retrospective study conducted between 2005 and 2013. Among 292,042 women of reproductive age, PHPT was present in 0.05%. Seventy-four women with untreated PHPT conceived, representing 0.03% of the entire cohort [2]. Two case series performed before 2000 reported on a total of 1600 parathyroidectomies, with 0.8 to 1.4% of them being carried out in pregnant women [3,4]. The diagnosis of PHPT in pregnancy can be confirmed in the presence of hypercalcemia (elevated serum ionized calcium or calcium adjusted for albumin) with a non-suppressed parathyroid hormone (PTH) level. PHPT is usually caused by a solitary parathyroid adenoma (~85%), hyperplasia is less frequent, and carcinoma is extremely rare with only eight cases reported in pregnancy [5,6,7,8,9,10,11,12]. In individuals below the age of 40 years, PHPT may be due to an underlying genetic mutation which occurs in approximately 10% of the cases [13]. Since pregnant women represent a considerably younger population, the presence of an underlying genetic mutation may be expected to be greater than 10%. Genetic mutations can present as part of a syndrome, such as multiple endocrine neoplasia (MEN 1, MEN2A or MEN4) or hyperparathyroidism-jaw tumor syndrome (HPT-JT), or it may be isolated as in familial isolated hyperparathyroidism (FIHP). Familial hypocalciuric hypercalcemia (FHH) should also be considered in the differential diagnosis of hypercalcemia along with a non-suppressed PTH. Appropriate evaluation is required prior to confirming the diagnosis of PHPT (see details in Section 4).

PHPT in pregnancy is associated with a number of maternal complications including hyperemesis gravidarum, nephrolithiasis and/or pancreatitis [14,15,16]. While neonatal hypocalcemia, tetany, intrauterine growth retardation (IUGR) and fetal demise have been previously reported with PHPT in pregnancy [17,18,19], milder forms of PHPT being diagnosed nowadays do not seem to carry the same degree of maternal or fetal mortality or morbidity [2,20]. Early recognition of PHPT has been associated with a lower rate of complications when compared to the older literature. However, medically managed PHPT still appears to be associated with an increased risk of preeclampsia and miscarriage rates [18,20]. The postpartum hypercalcemic crisis has been reported in the literature as a potential complication of PHPT in pregnancy, and this likely happens when the active transplacental transfer of calcium from the mother to the fetus is lost after delivery of the placenta [6,21,22].

In this review, we will present evidence-based practical guidance on the diagnosis and management of PHPT during pregnancy. Calcium homeostasis during pregnancy has been presented in detail in the accompanying article on hypoparathyroidism in pregnancy [23].

## 2. Materials and Methods

We carried out a literature search on MEDLINE, CINAHL, EMBASE and Google scholar databases from 1 January 1990 to 31 December 2020 using the following keywords: hyperparathyroidism; pregnancy; calcium homeostasis; parathyroidectomy; gestational hyperparathyroidism; cinacalcet; nephrolithiasis; preeclampsia; hypercalcemia. We reviewed 187 articles and included 110 in this review. These included review articles, clinical guidelines, book chapters, case reports and case series written in the English language. Letters to the editor were excluded from the review.

## 3. Impact of PHPT on Mother and Fetus during Pregnancy

Several maternal and fetal adverse outcomes have been associated with PHPT in pregnancy. Mothers can present with nephrolithiasis [16,24], hyperemesis gravidarum [15], and in severe cases acute pancreatitis [9,15,25,26,27,28,29,30]. Preeclampsia and hypertension have been frequently reported with PHPT in pregnancy [31,32,33,34,35]. PTH may stimulate the renin–angiotensin–aldosterone system and contribute to hypertension and preeclampsia [36,37,38,39,40,41]. Women with PHPT may develop a hypertensive crisis or HELLP syndrome which is characterized by hemolysis, elevated liver enzymes, low platelets and eclampsia or preeclampsia [38].

As for the non-pregnant population, PHPT may result in bone loss, especially at sites rich in cortical bone, such as the distal third radius, with relative sparing of the spine [42,43]. However, it should be noted that data pertaining to bone loss in pregnant women are limited as Dual-energy X-ray absorptiometry (DXA) assessment is seldom performed in such a population. In rare cases, fragility fractures may occur, and vertebral compression fractures, rib fractures as well as bilateral femur fractures have been reported in association with PHPT in pregnancy [44,45].

Neonatal hypocalcemia has been widely reported in the literature in association with maternal PHPT [46,47,48,49]. Other complications may include polyhydramnios [50], as well as fetal and maternal mortality [4,35,51].

An interesting case of twin pregnancy in a mother with PHPT was reported. The findings were discordant, with one of the babies presenting with neonatal hypocalcemia and a seizure, while the other was normocalcemic despite similar exposure to maternal hypercalcemia during pregnancy [52]. Therefore, it seems that the impact of maternal hypercalcemia on the developing fetus cannot be predicted by the degree of hypercalcemia alone.

Neonatal complications have been reported in women treated medically for PHPT throughout pregnancy [53]. Fetal mortality in medically treated pregnant women was estimated to be one in five fetuses (16%) while fetal mortality and morbidity in those who were treated surgically for PHPT were estimated to be 3% and 10%, respectively, based on data published from case reports from 1930 to 1990 [3]. Older literature is believed to represent more severe cases of PHPT in pregnancy with worse associated outcomes. Early recognition of PHPT with a milder degree of hypercalcemia has been associated with a lower rate of fetal and neonatal outcomes [20,54].

In another case series involving 17 pregnant women with PHPT, parathyroidectomy (PTX) was performed in the 2nd trimester with no maternal or fetal adverse outcomes and a 100% cure rate. One patient out of the 17 cases declined surgery during pregnancy and suffered from severe preeclampsia and delivered a baby with IUGR [17].

## 4. Diagnosis of PHPT in Pregnancy

**Figure 1:**
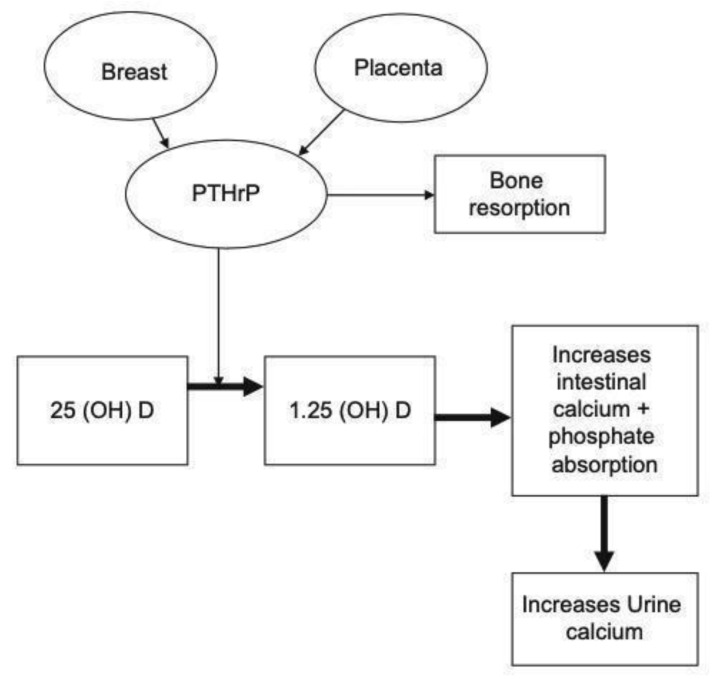
Calcium homeostasis during pregnancy (Reproduced with permission from Khan et al. EJE 2019) [59].

The diagnosis of PHPT is made in the presence of an elevated serum ionized calcium or calcium adjusted for albumin, with an elevated or non-suppressed PTH. During pregnancy, PTH levels may decline due to the physiological rise in PTHrP (Figure 1). PTHrP level rises as early as 3–13 weeks of gestation until it peaks in the third trimester [55,56,57,58], and PTH level may suppress as a result. Therefore, PTH levels may be lower than anticipated and the finding of hypercalcemia in the presence of a non-suppressed PTH level is suspicious for a diagnosis of PHPT during pregnancy. This must be taken into consideration when making the diagnosis of PHPT.

PHPT may occur as a sporadic parathyroid adenoma in the vast majority of cases, however, in individuals who present younger than 40 years old, an underlying genetic condition associated with the multiglandular disease should be excluded, see (Table 1) [60,61].

### 4.1. History

Nearly 80% of pregnant women with PHPT may be asymptomatic and are identified during routine lab testing for serum calcium levels [62]. It is, therefore, important to ask about symptoms of hypercalcemia when suspecting the diagnosis of PHPT, as they may overlap with the normal physiological changes seen during pregnancy. These symptoms include lethargy, weakness, nausea, vomiting, polyuria, and polydipsia. Other symptoms of hypercalcemia may also be present and include constipation, abdominal or epigastric pain, depression, and confusion. Some women may even present with hyperemesis gravidarum, acute pancreatitis, nephrolithiasis, and preeclampsia [6,21,22,31,61,62,63,64]. After evaluating for symptoms of hypercalcemia, it is essential to assess for prior history of fragility fractures or height loss due to vertebral fractures, which may be silent. Further, a history of previous kidney stones should be obtained.

It is also essential to determine if the PHPT is isolated or presents as part of a syndrome. The possibility of MEN syndromes, as well as FHH, must be excluded before confirming the diagnosis of PHPT and prior to considering the patient for surgical intervention. Current use of medications or supplements such as calcium supplements, lithium, or hydrochlorothiazide should be excluded. Family history of parathyroid disease, MEN and FHH should also be addressed. In the setting of hypercalcemia, it is crucial to exclude other underlying conditions that can cause hypercalcemia, namely granulomatous disease, occult malignancy or adrenal insufficiency [60,65]. Hypercalcemia resulting from these conditions is non-PTH-mediated and therefore, PTH is expected to be suppressed.

### 4.2. Physical Examination

General physical examination includes measurement of blood pressure, pulse, height and weight. Carefully palpate the thyroid gland for nodules. Examine for neck scars and evidence of prior neck surgery. Chest, cardiovascular, breast and abdominal examination must be completed in order to search for signs of an occult malignancy if PTH levels are mid normal or suppressed [60]. Assessment of fetal well-being as per recommended guidelines is advised to be completed by the treating obstetrician.

### 4.3. Laboratory Investigations

Measure serum ionized calcium, calcium adjusted for albumin (see Formula (1)), intact PTH, phosphorus, magnesium, creatinine, estimated GFR, 25 hydroxyvitamin D (25(OH_2_)D_3_), TSH, free T4, free T3, full blood count and alkaline phosphatase. Proceed with 24-h urine collections for calcium and creatinine, with the calculation of the calcium to creatinine clearance ratio (CCCR) for the exclusion of FHH (see Formula (2)).

1. Calcium adjusted for Albumin Formula.

Corrected calcium (mmol/L) = measured total calcium + (40 − serum albumin) × 0.02 Corrected calcium (mg/dL) = measured total Ca(mg/dL) + 0.8 (4.0 − serum albumin (g/dL))(1)

2. Calculations of Calcium to Creatinine Clearance Ratio (CCCR).

(2) $$\frac{\mathrm{Urine}\;\mathrm{Calcium}\;\left(\mathrm{mmol}/L\right)\;\times \;\left[\mathrm{Serum}\;\mathrm{Creatinine}\;\left(\mathrm{umol}/L\right)/1000\right]}{\mathrm{Serum}\;\mathrm{Calcium}\;\left(\mathrm{mmol}/L\right)\;\times \;\mathrm{Urine}\;\mathrm{Creatinine}\;\left(\mathrm{mmol}/L\right)}$$

The coexistence of vitamin D inadequacy may cause secondary elevations in PTH level and this is not uncommon for patients with PHPT. The prevalence of vitamin D inadequacy has been shown to be higher amongst non-pregnant patients with PHPT than in the general population. High PTH increases the conversion of 25(OH_2_)D_3_ into active 1.25(OH_2_)D_3_ and also leads to vitamin D inactivation through enhanced hepatic metabolism [66]. Therefore, it is recommended to correct coexisting vitamin D inadequacy prior to confirming the diagnosis of PHPT and aim for 25(OH_2_)D_3_ level between 50 and 125 nmol/L (20–50 ng/mL) [1,67,68,69].

Due to the presence of absorptive hypercalciuria in pregnancy, CCCR requires careful interpretation [70]. Absorptive hypercalciuria is better demonstrated on a 24-h urine sample, as fasting spot urine calcium may remain low despite significant 24-h urine hypercalciuria. Therefore, the use of spot urine calcium is not advised as the result may be misleading [71].

In 80% of non-pregnant individuals with FHH, CCCR is expected to be less than 0.01, however, in 20% of cases, it is found to be between 0.01 and 0.02 [72]. If the calculated CCCR during pregnancy is low (less than 0.01), this is of great value in excluding PHPT, and DNA analysis of the calcium-sensing receptor gene (*CaSR*) can be completed in order to obtain a molecular diagnosis of FHH. There have been four reported cases of pregnant women with FHH, and in these women, CCCR was higher than anticipated with the highest value being up to 0.019 [73].

Consider preeclampsia panel when clinically indicated. If an underlying genetic disorder is suspected, further investigations may be required (see Table 1).

### 4.4. Localization

Imaging should only be considered if the patient is referred for surgery. Imaging during pregnancy is best limited to neck ultrasound which has a sensitivity of 76% to 87% in identifying abnormal parathyroid tissue and a specificity between 94% and 96% [74,75,76]. The sensitivity of ultrasound is lower in multiglandular disease and may be only 15–35% [77,78]. Magnetic resonance imaging (MRI) can be used [79], however, it has a sensitivity of 82% [80] in detecting parathyroid adenoma and should not be used as a single imaging modality in localizing parathyroid adenoma [81]. MRI with gadolinium contrast should be limited in pregnancy since this agent is water-soluble and may cross the placenta [82]. Radiation exposure should be avoided during pregnancy due to the increased risk of fetal anomalies or death, intellectual disability, or subsequent cancer from the ionizing radiation [83,84], therefore the use of computed tomography (CT) and 99mTc Sestamibi should be avoided in pregnancy.

## 5. Clinical Management of PHPT in Pregnancy

There is no consensus on the management of PHPT during pregnancy, however, an individualized approach is required for women with PHPT. This approach is dependent on the severity of symptoms, gestational age at presentation, age, associated features, and complications. Mild PHPT (serum corrected calcium less than 2.85 mmol/L (11.42 mg/dL)) in pregnancy may be managed conservatively.

Medical management includes adequate hydration and cessation of thiazide diuretics, calcium supplements and lithium if possible [85,86]. Pharmacologic options are severely limited with no safety data with any of the available treatment strategies.

Calcitonin is reserved for refractory hypercalcemia. It is classified as category C in pregnancy and it does not cross the placenta [87]. It has been used in a number of case reports [85,88]. Associated tachyphylaxis may limit its long-term efficacy.

Calcimemetics, namely cinacalcet, increases the sensitivity of the parathyroid CaSR to extracellular calcium, resulting in a reduction in PTH secretion [89]. It is categorized as class C in pregnancy and has been shown to cross the placenta. Data on long-term safety in pregnancy are unfortunately lacking. There are six published case reports describing cinacalcet use during pregnancy in patients with PHPT [5,24,85,88,90,91]. Neonatal hypocalcemia was reported in three out of the six cases treated with cinacalcet [5,24,88]. This, however, may be in association with maternal hypercalcemia and a causal relationship with cinacalcet has not been confirmed. Cinacalcet has also been used in a pregnant woman with PHPT due to parathyroid carcinoma with no reported maternal or fetal unfavorable outcomes [5].

Bisphosphonates should be avoided during pregnancy as they cross the placenta and adverse fetal skeletal outcomes have been observed in animal studies [92]. Similarly, denosumab is classified as category D in pregnancy, it crosses the placenta and has been associated with skeletal fetal adverse outcomes in animal studies and should be avoided in pregnancy [93,94].

Oral phosphate has been previously used to lower serum calcium levels, however, safety and efficacy data during pregnancy are very limited and use should not be recommended [62,95,96,97].

Surgery remains the only curative option for PHPT. It is well-tolerated during pregnancy and adverse events are minimal [44]. Timely surgical intervention in the 2nd trimester has been associated with favorable outcomes in patients with moderate to severe hypercalcemia during pregnancy (calcium adjusted for albumin greater than 3 mmol/L (12.02 mg/dL)) [98,99]. This has shown to be an effective approach in several case reports and case series [17,29,32,100,101,102].

Surgical intervention in the 3rd trimester of pregnancy has been reported in patients who have failed medical therapy. In the majority of these cases, surgery was safe and did not result in any harmful maternal or fetal outcomes [21,31,99,100,103,104,105,106,107,108,109]. Some cases, however, reported associated preeclampsia, preterm labor and severe neonatal hypocalcemia with surgery in the 3rd trimester [21,35,110,111]. These complications may be attributed to delayed presentation and prolonged exposure to maternal hypercalcemia. Parathyroidectomy has also been performed successfully in the 1st trimester [112].

Intraoperative PTH measurement is recommended to confirm successful resection of the hyperfunctioning parathyroid adenoma [1]. It can be of a great value in pregnant women in whom preoperative localization of abnormal parathyroid tissue is not conclusive or in those with the multiglandular disease.

## 6. Conclusions

In conclusion, PHPT in pregnancy can be associated with adverse fetal and maternal outcomes if not carefully managed. Published literature is limited to case series and case reports. In women of childbearing age with PHPT identified prior to pregnancy, it is critically essential to arrange for a preconception counselling and proceed with a definitive surgery based on the underlying etiology of PHPT prior to conception.

In women with mildly elevated serum ionized calcium or calcium adjusted for albumin, we suggest adequate hydration as well as close monitoring of the serum calcium (ionized or corrected). If calcium adjusted for albumin is greater than 3 mmol/L (12.02 mg/dL), surgical intervention in the 2nd trimester is advised. Surgical intervention with PTX by experienced endocrine surgeons has been shown to be safe, curative and effective. Wide evidence supports PTX timed in the 2nd trimester, however, several case reports confirmed the safety of PTX in the 1st and 3rd trimesters [21,31,99,100,103,104,105,106,107,108,109].

If conservative management is chosen during pregnancy, women need to be monitored closely throughout pregnancy and in the postpartum period, as the maternal–fetal calcium transfer will be interrupted after delivery of the placenta and this may result in a hypercalcemic crisis.

Our suggestions are based on very low-quality evidence. A multidisciplinary approach with close communication amongst the endocrinologist, obstetrician and pediatrician is recommended for optimal maternal and fetal outcomes.

## Figures and Tables

**Table 1:** Causes and Diagnostic Tools for PHPT.

Genetic Conditions (Consider If Age Is Less than 40 Years)	MEN1	MEN2A	MEN4	HPT-JT	Exclude FHH
Associated conditions/Features	Parathyroid adenoma/hyperplasia Enteropancreatic cell tumors Anterior pituitary lesion carcinoid tumors, adrenocortical tumors, meningiomas,	Parathyroid adenoma/hyperplasia MTC Pheochromocytoma	Parathyroid Pituitary Adrenals Kidneys Gonadal tumors	Parathyroid, uterine, testicular, renal tumors ossifying jaw fibroma, pancreatic adenocarcinoma Thyroid (Hurthle cell adenoma)	Family history of hypercalcemia CCCR < 0.02
Primary investigations	Primary panel: serum ionized calcium, calcium adjusted for albumin, intact PTH, phosphorus, magnesium, creatinine, eGFR, 25(OH_2_)D_3_, TSH, freeT4, freeT3, Full blood count, alkaline phosphatase. +/− LH, FSH, PRL, estradiol, IGF1 (performed outside of pregnancy) ACTH *, cortisol *, gastrin, glucagon, chromogranin A, vasointestinalpolypeptide, pancreatic polypeptide, insulin, fasting glucose level	Primary panel +/− Calcitonin, plasma metanephrines, 24 h urinary metanephrines	Primary panel	Primary panel	Primary panel Gene sequencing FHH type1–*CaSR* inactive mutation FHH type2–*GNA11* mutation FHH type3–*AP2S1* gene mutation
Confirmatory Test	*MEN1* gene analysis	*RET* protooncogene analysis	*CDNK1B* gene sequencing	*CDC73* gene sequencing (parafibromin)	

Abbreviations: MEN1, 2A, 4, multiple endocrine neoplasia; HPT-JT, hyperparathyroidism jaw tumor syndrome; CCCR, calcium to creatinine clearance ratio; *CaSR*, calcium-sensing receptor gene; MTC, medullary thyroid cancer; +/−: with or without based on clinical presentation; * please note values are altered during pregnancy secondary to the physiological changes.

## Data Availability

No new data were created or analyzed in this study.
